# Computational simulation of a new system modelling ions electromigration through biological membranes

**DOI:** 10.1186/1742-4682-10-51

**Published:** 2013-09-05

**Authors:** Noureddine Alaa, Hamid Lefraich

**Affiliations:** 1 Department of Mathematics, Laboratory of Applied Mathematics and Computer Science (LAMAI), Faculty of Science and Technology, Cadi Ayaad University, Abdelkarim Elkhattabi Avenue, Marrakech, Morocco

**Keywords:** Reaction-diffusion system, Electromigration, Nonlinear coupled system, Finite element method, Nernst-Planck equations, Numerical analysis, Enzyme kinetics, Substrate suicide, Cooperative phenomena, Computational simulation

## Abstract

**Background:**

The interest in cell membrane has grown drastically for their important role as controllers of biological functions in health and illness. In fact most important physiological processes are intimately related to the transport ability of the membrane, such as cell adhesion, cell signaling and immune defense. Furthermore, ion migration is connected with life-threatening pathologies such as metastases and atherosclerosis. Consequently, a large amount of research is consecrated to this topic. To better understand cell membranes, more accurate models of ionic flux are required and also their computational simulations.

**Results:**

This paper is presenting the numerical simulation of a more general system modelling ion migration through biological membranes. The model includes both the effects of biochemical reaction between ions and fixed charges. The model is a nonlinear coupled system. In the first we describe the mathematical model. To realize the numerical simulation of our model, we proceed by a finite element discretisation and then by choosing an appropriate resolution algorithm to the nonlinearities.

**Conclusions:**

We give numerical simulations obtained for different popular models of enzymatic reaction which were compared to those obtained in literature on systems of ordinary differential equations. The results obtained show a complete agreement between the two modellings. Furthermore, various numerical experiments are presented to confirm the accuracy, efficiency and stability of the proposed method. In particular, we show that the scheme is unconditionally stable and second-order accurate in space.

## Background

Cell membrane is the biological membrane separating the intracellular environment from the extracellular one. The cell membrane surrounds all cells and it’s selectively permeable, permitting the free passage of some substances and restricting the passage of others, thus controlling the flux of substances in and out of the cell. All diseases are problems of regulating the passage of materials at the level of the cell. Consequently, to understand the cause of a disease, we need to understand the alterations that take place at the cellular level. Thanks to mathematical modeling, cell biology phenomena may be expressed by ordinary differential equations or systems of partial differential equations.

An important class of models in cell biology, is the class modeling ion transport through biological membranes. This transport phenomena occurs in most living cells and some biochemical processes. The first models in the literature included one ordinary differential equation for each ion concentration. All this models were based on the implicit assumptions that chemical concentrations are uniform in space. This assumption is reasonable when the region of space where the reaction occurs is confined and very small. Also this models assumed that the electric field is constant inside the membrane.

Despite that the constant electrical field assumption has the advantage of leading to a simple mathematical analysis, all cells maintain a difference in electrical charge across their membrane. This difference in charge give rise to a voltage difference, or electrical potential. Furthermore, there are numerous situations in which chemical concentrations are nonuniform in space. In this sense, we need to establish and compute more accurate mathematical models of ions electromigration through biological membranes.

In this paper, we present the numerical simulation of a more realistic model of ions electromigration through biological membranes. This model is more general than those in literature of membrane transport as it extends them in four topics: 1) it’s a multidimensional model, 2) it doesn’t rely on the constant electrical field assumption, 3) it consider both the temporal and spatial dependence, 4) it includes different reaction kinetics terms.

## Introduction

In this paper we consider a class of models of ions migration through biological membranes. Such migrations exist for most living cells and some biochemical processes. The motion of ions is supposed due to diffusion and to the effect of the electrical field. Furthermore ions can undergo reactions. So the ions concentrations satisfy the Nernst-Planck equations, including a kinetic reaction terms and the potential is given by Poisson equation. The model is given by:

(1) $$\left\{\begin{array}{l} \;\frac{\partial C_{i}}{\mathrm{\partial t}}-d_{i}\Delta C_{i}-m_{i}\text{div}(C_{i}\nabla \phi )=F_{i}(C_{1},\ldots ,C_{N_{s}})\;\text{in}\;Q_{T},\text{for}i=1,\ldots ,\text{Ns}\\ \;-\varepsilon \Delta \phi =\overset{N_{s}}{\underset{i=1}{\sum }}z_{i}C_{i}-f\;\text{in}\;Q_{T}\\ \;d_{i}\frac{\partial C_{i}}{\partial \upsilon }+m_{i}C_{i}\frac{\partial \phi }{\partial \upsilon }=0\;\text{in}\;\sum_{T},\text{for}i=1,\ldots ,\text{Ns}\\ \;\phi (t,x)=0\;\text{in}\;\sum_{T}\\ C_{i}(0,x)=C_{i,0}(x)\;\text{on}\;\Omega \\ \;\phi (0,x)=\phi_{0}(x)\;\text{on}\;\Omega \end{array}\right)$$

where *Q*_*T*_=]0,*T*[×*Ω*, $\sum_{T}=]0,T[\times \partial \Omega$, *T*>0; *Ω* is an open regular set of ${\mathbb{R}}^{2}$ which represent the biological cell and *∂Ω* represent the cell membrane.

For each *i*,*C*_*i*_ is the concentration of the *i* species which has diffusion coefficient *d*_*i*_, mobility *m*_*i*_ and valency *z*_*i*_. *ϕ* is the electrical potential, *f* is the fixed charges concentration and the *F*_*i*_ are reaction terms. We suppose that *F*_*i*_ depends continuously on the *C*_*j*_ ’s and *ϕ*, and that *f* is a bounded function. We suppose that *d*_*i*_ is a positive constant for each *i*.

(2) $$\text{for all}i=1,\ldots ,\text{Ns}\;C_{i,0}\in L^{2}(\Omega )\text{and satisfy}C_{i,0}\geq 0$$

Further information about the modelling of the problem and its mathematical analysis, can be found in [1].

In the biophysical literature, the early works on these models were interested in the stationary case of passive migration (i.e., without reaction); see [2,3]. In all these works two popular simplifications were considered, namely the Goldman hypothesis where the electrical field is supposed to be constant inside the membrane and the electroneutral hypothesis where the neutrality at each point of the membrane is assumed; see for example [3]. Mcgillivray [4] recognized that these models are the limit of the full equations when the ratio $\sqrt{\varepsilon }=\frac{\lambda }{l}$ of the Debye length to the membrane thickness goes to, respectively, infinity or zero. Usually enzymes are held to biological membranes and ions undergo biochemical reactions when crossing the membrane. Valleton [5] did a general biophysical study of coupling of electromigration diffusion with biochemical reactions.

In this paper we present a numerical simulation of such systems, for a large class of reaction kinetics, including the usual biochemical kinetics as the Michaelis-Menton one (a mathematical analysis of the one dimensional and stationary case was done by [6] then we did the mathematical analysis of the multidimensional unsteady case [1]). This article is organized in the following way. The next section is devoted to finite element discretisation of the mathematical model. Then, we present applications, results and numerical experiments showing the accuracy, efficiency and stability of the proposed method. Finally, conclusions are drawn in the last section.

## Finite element discretisation

In order to show the numerical formulation of the problem, let *V* = *L*^2^(0,*T*;*H*^1^(*Ω*)) be the space of approximate solutions and *W* = *H*^1^(*Ω*) be the space of tests functions. Let *W*^*h*^ be a finite element space of Lagrange *P*1 included in *W* and *V*^*h*^=*L*^2^(0,*T*;*W*^*h*^) be the finite dimensional subspace of *V*. Now we introduce the function $Z_{h}={\left(Z_{i,h}\right)}_{1\leq i\leq N_{s}}$ defined by

$$Z_{i,h}=C_{i,h}\;\text{exp}\;\left(\frac{m_{i}}{d_{i}}(\phi_{h})\right)\;\text{for}i=1,\ldots ,N_{s}$$

Moreover, we consider

$$p_{i,h}=\;\text{exp}\;\left(\frac{m_{i}}{d_{i}}\phi_{h}\right)\;\text{and}\;q_{i,h}=\frac{1}{p_{i,h}}$$

The Faedo-Galerkin formulation for the problem is given by, finding *Z*_*i*,*h*_∈*V*_*h*_ for *i* = 1,…,*N*_*s*_*andϕ*_*h*_∈*V*_*h*_ such that *ϕ*_*h*_=0 in *∂Ω*:

(3) $$\left\{\begin{array}{l} ●\;\text{for every}\;w_{h}\in W_{h}\text{a.e. t}\in ]0,T[\;\text{and for}\;1\leq i\leq N_{S},\\ \frac{d}{\text{dt}}\int_{\Omega }q_{i,h}Z_{i,h}w_{h}+\int_{\Omega }d_{i}q_{i,h}\nabla Z_{i,h}\nabla w_{h}=\int_{\Omega }\tilde{F_{i}}(Z_{h})w_{h}\\ Z_{i,h}(0)=q_{i,h}(0)C_{i,0}^{h},\\ ●\;\text{for all}\;v_{h}\in W_{h}\;\text{such that}\;v_{h}=0\text{in}\partial \Omega \;\text{and a.e. t}\;\in ]0,T[\\ \varepsilon \int_{\Omega }\nabla \phi_{h}\nabla v_{h}=\int_{\Omega }(\overset{N_{s}}{\underset{j=1}{\sum }}z_{j}q_{j,h}Z_{j,h}-f)v_{h}\\ \phi_{h}(0)=\phi_{0}^{h}\;\text{on}\;\Omega \end{array}\right)$$

where ${\tilde{F}}_{i}(X_{1},\ldots ,X_{N_{s}})=F_{i}(q_{1,h}X_{1},\ldots ,q_{N_{s},h}X_{N_{s}})$ and $C_{i,0}^{h},\;\phi_{0}^{h}$ are the projections of *C*_*i*,0_, *ϕ*_0_ on *W*_*h*_.Let (*y*_*j*_)_1≤*j*≤*m*_ the mesh nodes, (*Φ*_*j*_)_1≤*j*≤*m*_ the canonical basis of *W*_*h*_, we consider the following two sets of index

$$\begin{array}{l} Y_{0}\;=\;\left\{j\in \left[1:m\right],\;y_{j}\in \partial \Omega \right\}\\ Y\;=\;\left[1:m\right]-Y_{0} \end{array}$$

We represent the solutions as $Z_{i_{s},h}(t,x)=\overset{m}{\underset{j=1}{\sum }}\alpha_{Z_{i_{s},j}}(t)\Phi_{j}(x)$ for *i*_*s*_=1,…,*N*_*s*_ and $\phi_{h}(t,x)=\overset{m}{\underset{j=1}{\sum }}\alpha_{j}(t)\Phi_{j}(x)$ with *α*_*j*_(*t*)=0 for *j*∈*Y*_0_. Then we set $\xi_{Z_{i_{s}}}=\xi_{Z_{i_{s}}}(t)={\left(\alpha_{Z_{i_{s}},j}(t)\right)}_{1\leq j\leq m}$ for *i*_*s*_=1,…,*N*_*s*_ and *ζ*_*ϕ*_=*ζ*_*ϕ*_(*t*)=(*α*_*j*_(*t*))_1≤*j*≤*m*_.

Now let’s consider an uniform subdivision of [0,*T*], we define a time step $\text{dt}=\frac{T}{N},\;$for *N*≥1. We pose then:

$$t_{n}=\text{ndt},\;0\leq n\leq N$$

Let’s note $\xi_{Z_{i_{s}}^{n}}$ the approximation of $\xi_{Z_{i_{s}}}\left(t_{n}\right)$ and $\zeta_{\phi^{n}}$ the approximation of *ζ*_*ϕ*_(*t*_*n*_), then we used an implicit scheme for the discretization of the time derivative. By the method of finite elements see [7], we arrive at the following formulation of the problem: Find the vectors nodal concentrations $\xi_{Z_{i_{s}}^{n+1}}={\left(\alpha_{Z_{i_{s}}^{n+1},j}\right)}_{1\leq j\leq m}$ for every 1≤*i*_*s*_≤*N*_*s*_ and nodal potential $\zeta_{\phi^{n+1}}={\left(\alpha_{j}^{n+1}\right)}_{1\leq j\leq m}\;\text{with}\;\alpha_{j}^{n+1}=0$ for *j*∈*Y*_0_ such that

$$\;\left\{\;\;\begin{array}{l} \frac{1}{\text{dt}}\;\left(\;A_{i_{s},\zeta_{\phi^{n+1}}}\xi_{Z_{i_{s}}^{n+1}}\;-\;A_{i_{s},\zeta_{\phi^{n}}}\xi_{Z_{i_{s}}^{n}}\;\right)\;+\;R_{i_{s},\zeta_{\phi^{n+1}}}\xi_{Z_{i_{s}}^{n+1}}\;=\;S_{i_{s},\zeta_{\phi^{n+1}}}\;(\xi_{Z_{1}^{n+1}},\;\ldots \;,\xi_{Z_{N_{s}}^{n+1}})\;\text{for}\;i_{s}\;=\;1,\;\ldots \;,N_{s}\\ \varepsilon Q_{Y\times Y}{\left(\zeta_{\phi^{n+1}}\right)}_{Y}=\overset{N_{s}}{\underset{i_{s}=1}{\sum }}{\left(z_{i_{s}}M_{i_{s},\zeta_{\phi^{n}}}\right)}_{Y\times Y}{\left(\xi_{Z_{i_{s}}^{n}}\right)}_{Y}-\beta_{Y}\\ \xi_{Z_{i_{s}}^{0}}=\xi_{Z_{i_{s}}}(0),\;\zeta_{\phi^{0}}=\zeta_{\phi }(0) \end{array}\right)$$

Let *T*_*h*_ the mesh generation of *Ω* containing *nel* finite elements. For a finite element *e*_*k*_∈*T*_*h*_, let be $T^{e_{k}}=\left\{k_{1},\;k_{2},\;k_{3}\right\}$ where *k*_1_, *k*_2_, *k*_3_ are the numbers of degrees of freedom of *e*_*k*_ and $N_{k_{l}}^{e_{k}}$ are interpolation functions. We have $\Phi_{i}{/}_{e_{k}}=N_{k_{s}}^{e_{k}},\;\Phi_{j}{/}_{e_{k}}=N_{k_{l}}^{e_{k}}$ where *s*,*l*∈{1, 2, 3} if and only if $\left(i,\;j\right)\in T^{e_{k}}\times T^{e_{k}}.$Where $A_{i_{s},\zeta_{\phi^{n}}}={\left(a_{\text{ij}}^{i_{s},n}\right)}_{1\leq i,j\leq m}$, $R_{i_{s},\zeta_{\phi^{n}}}=\;{\left(r_{\text{ij}}^{i_{s},n}\right)}_{1\leq i,j\leq m}$, $\;S_{i_{s},\zeta_{\phi^{n+1}}}\left(\xi_{Z_{1}^{n+1}},\ldots ,\xi_{Z_{N_{s}}^{n+1}}\right)={\left(s_{i}^{i_{s},n+1}\right)}_{1\leq i\leq m}$ for *i*_*s*_=1,…,*N*_*s*_, *Q* = (*q*_*ij*_)_1≤*i*,*j*≤*m*_, $M_{i_{s},\zeta_{\phi^{n}}}={\left(m_{\text{ij}}^{i_{s},n}\right)}_{1\leq i,j\leq m}$, *β* = (*β*_*i*_)_1≤*i*≤*m*_(*X*_*Y*×*Y*_ means the extracted matrix from *X* by keeping lines and columns with numbers belonging to *Y*)

$$\overset{i_{s},n}{\underset{\text{ij}}{a}}=\overset{\text{nel}}{\underset{k=1}{\sum }}\int_{e_{k}}\;\text{exp}\;\left(\frac{-m_{i_{s}}}{d_{i_{s}}}\underset{l=1}{\overset{m}{\sum }}\alpha_{l}^{n}{\Phi_{l}}_{|e_{k}}\right){\Phi_{j}}_{|e_{k}}{\Phi_{i}}_{|e_{k}}=\overset{\text{nel}}{\underset{k=1}{\sum }}a_{\text{ij}}^{i_{s},n,e_{k}}$$

where

$$\begin{array}{ll} a_{\text{ij}}^{i_{s},n,e_{k}} & =\left\{\begin{array}{ll} \int_{e_{k}}\;\text{exp}\;\left(\frac{-m_{i_{s}}}{d_{i_{s}}}\underset{p=1}{\overset{3}{\sum }}\alpha_{k_{p}}^{n}N_{k_{p}}^{e_{k}}\right)N_{k_{l}}^{e_{k}}N_{k_{s}}^{e_{k}} & \;\text{if}\;\left(i,\;j\right)\in T^{e_{k}}\times T^{e_{k}}\\ \;0\; & \;\text{if}\;i\text{or}j\notin T^{e_{k}} \end{array}\right)\\ r_{\text{ij}}^{i_{s},n} & =\overset{\text{nel}}{\underset{k=1}{\sum }}d_{i_{s}}\int_{e_{k}}\;\text{exp}\;(\frac{-m_{i_{s}}}{d_{i_{s}}}\underset{l=1}{\overset{m}{\sum }}\alpha_{l}^{n}{\Phi_{l}}_{|e_{k}}))\nabla {\Phi_{j}}_{|e_{k}}\nabla {\Phi_{i}}_{|e_{k}}=\overset{\text{nel}}{\underset{k=1}{\sum }}r_{\text{ij}}^{i_{s},n,e_{k}} \end{array}$$

where

$$\;r_{\text{ij}}^{i_{s},n,e_{k}}\;=\;\left\{\begin{array}{l} d_{i_{s}}\int_{e_{k}}\;\text{exp}\;\left(\frac{-m_{i_{s}}}{d_{i_{s}}}\underset{p=1}{\overset{3}{\sum }}\alpha_{k_{p}}^{n}N_{k_{p}}^{e_{k}}\right)\nabla N_{k_{l}}^{e_{k}}\nabla N_{k_{s}}^{e_{k}}\;\text{if}\;\left(i,j\right)\in T^{e_{k}}\times T^{e_{k}}\\ \;0\;\text{if}\;i\;\text{or}\;j\notin T^{e_{k}} \end{array}\right)$$

$$\begin{array}{l} s_{i}^{i_{s},n+1}\;=\;\sum_{k=1}^{\text{nel}}\int_{e_{k}}F_{i_{s}}\left(\;\text{exp}\;\left(\frac{-m_{1}}{d_{1}}\underset{l=1}{\overset{m}{\sum }}\alpha_{l}^{n+1}{\Phi_{l}}_{|e_{k}}\right)\overset{m}{\underset{j=1}{\sum }}\alpha_{Z_{1}^{n+1},j}{\Phi_{j}}_{|e_{k}},\ldots ,\right)\\ \;\left(\;\text{exp}\;\left(\frac{-m_{N_{s}}}{d_{N_{s}}}\underset{l=1}{\overset{m}{\sum }}\alpha_{l}^{n+1}{\Phi_{l}}_{|e_{k}}\right)\overset{m}{\underset{j=1}{\sum }}\alpha_{Z_{N_{s}}^{n+1},j}{\Phi_{j}}_{|e_{k}}\right){\Phi_{i}}_{|e_{k}}=\overset{\text{nel}}{\underset{k=1}{\sum }}s_{i}^{i_{s},n+1,e_{k}} \end{array}$$

where

$$\;\begin{array}{l} s_{i}^{i_{s},n+1,e_{k}}\;=\;\left\{\begin{array}{l} \int_{e_{k}}F_{i_{s}}\;\left(\;\text{exp}\;\left(\;\frac{-m_{1}}{d_{1}}\underset{p=1}{\overset{3}{\sum }}\alpha_{k_{p}}^{n+1}N_{k_{p}}^{e_{k}}\right)\overset{3}{\underset{l=1}{\sum }}\alpha_{Z_{1}^{n+1},k_{l}}N_{k_{l}}^{e_{k}},\ldots ,\;\text{exp}\;\left(\frac{-m_{N_{s}}}{d_{N_{s}}}\underset{p=1}{\overset{3}{\sum }}\alpha_{k_{p}}^{n+1}N_{k_{p}}^{e_{k}}\right)\overset{3}{\underset{l=1}{\sum }}\alpha_{Z_{N_{s}}^{n+1},k_{l}}N_{k_{l}}^{e_{k}}\right)N_{k_{s}}^{e_{k}}\;\text{if}\;i\in T^{e_{k}}\\ \;0\;\text{if}\;i\notin T^{e_{k}} \end{array}\right)\\ \;q_{\text{ij}}=\overset{\text{nel}}{\underset{k=1}{\sum }}\int_{e_{k}}\nabla {\Phi_{j}}_{|e_{k}}\nabla {\Phi_{i}}_{|e_{k}}=\overset{\text{nel}}{\underset{k=1}{\sum }}q_{\text{ij}}^{e_{k}} \end{array}$$

where

$$\;\;\;q_{\text{ij}}^{e_{k}}\;=\;\left\{\begin{array}{l} \int_{e_{k}}\nabla N_{k_{l}}^{e_{k}}\nabla N_{k_{s}}^{e_{k}}\;\text{if}\;\left(i,\;j\right)\in T_{e_{k}}\times T_{e_{k}}\\ \;0\;\text{if}\;i\;\text{or}\;j\notin T^{e_{k}} \end{array}\right)$$

$$m_{\text{ij}}^{i_{s},n}=\overset{\text{nel}}{\underset{k=1}{\sum }}\int_{e_{k}}\;\text{exp}\;\left(\frac{-m_{i_{s}}}{d_{i_{s}}}\underset{l=1}{\overset{m}{\sum }}\alpha_{l}^{n}{\Phi_{l}}_{|e_{k}}\right){\Phi_{j}}_{|e_{k}}{\Phi_{i}}_{|e_{k}}=\overset{\text{nel}}{\underset{k=1}{\sum }}m_{\text{ij}}^{i_{s},n,e_{k}}$$

where

$$\begin{array}{l} m_{\text{ij}}^{i_{s},n,e_{k}}=\left\{\begin{array}{l} \int_{e_{k}}\;\text{exp}\;\left(\frac{-m_{i_{s}}}{d_{i_{s}}}\underset{p=1}{\overset{3}{\sum }}\alpha_{k_{p}}^{n}N_{k_{p}}^{e_{k}}\right)\overset{e_{k}}{\underset{k_{l}}{N}}N_{k_{s}}^{e_{k}}\text{if}\left(i,\;j\right)\in T_{e_{k}}\times T_{e_{k}}\\ \;0\;\text{if}\;i\;\text{or}\;j\notin T^{e_{k}} \end{array}\right)\\ \;\beta_{i}=\overset{\text{nel}}{\underset{k=1}{\sum }}\int_{e_{k}}f\;N_{k_{s}}^{e_{k}}=\overset{\text{nel}}{\underset{k=1}{\sum }}\beta_{i}^{e_{k}} \end{array}$$

where

$$\beta_{i}^{e_{k}}=\left\{\begin{array}{l} \int_{e_{k}}f\;N_{k_{s}}^{e_{k}}\;\text{if}\;i\in T^{e_{k}}\\ \;0\;\text{if}\;i\notin T^{e_{k}} \end{array}\right)$$

Finally, finding $\xi_{Z_{i_{s}}^{n+1}}={\left(\alpha_{Z_{i_{s}}^{n+1},j}\right)}_{1\leq j\leq m}$ for every 1≤*i*_*s*_≤*N*_*s*_ and $\zeta_{\phi^{n+1}}={\left(\alpha_{j}^{n+1}\right)}_{1\leq j\leq m}\;\text{with}\;\alpha_{j}^{n+1}=0$ for *j*∈*Y*_0_ such that

$$\;\left\{\begin{array}{l} \left(A_{i_{s},\zeta_{\phi^{n+1}}}+{\text{dtR}}_{i_{s},\zeta_{\phi^{n+1}}}\right)\xi_{Z_{i_{s}}^{n+1}}=A_{i_{s},\zeta_{\phi^{n}}}\xi_{Z_{i_{s}}^{n}}+{\text{dtS}}_{i_{s},\zeta_{\phi^{n+1}}}\left(\xi_{Z_{1}^{n+1}},\ldots ,\xi_{Z_{N_{s}}^{n+1}}\right)\;\text{for}\;i_{s}=1,\ldots ,N_{s}\\ \varepsilon Q_{Y\times Y}{\left(\zeta_{\phi^{n+1}}\right)}_{Y}=\overset{N_{s}}{\underset{i_{s}=1}{\sum }}{\left(z_{i_{s}}M_{i_{s},\zeta_{\phi^{n}}}\right)}_{Y\times Y}{\left(\xi_{Z_{i_{s}}^{n}}\right)}_{Y}-\beta_{Y}\\ \xi_{Z_{i_{s}}^{0}}=\xi_{Z_{i_{s}}}(0),\;\zeta_{\phi^{0}}=\zeta_{\phi }(0) \end{array}\right)$$

We have a nonlinear term due to $S_{i_{s},\zeta_{\phi^{n+1}}}(\xi_{Z_{1}^{n+1}},\ldots ,\xi_{Z_{N_{s}}^{n+1}})$, we have dealt with according to the model and thus to the expression of $S_{i_{s},\zeta_{\phi^{n+1}}}$.

## Results and discussion

In this section we present three numerical applications of ions electro-migration through biological membranes. The models of the basic enzyme reaction, the suicide substrate reaction and the cooperative reaction, are numerically simulated.

### Result 1 : Enzyme kinetics (basic enzyme reaction)

To understand where some of the more complicated reaction schemes come from, we consider a reaction that is catalyzed by an enzyme. Enzymes act as remarkably efficient catalysts (generally proteins), by accelerating the conversion of some other molecules called substrates into products via lowering the free energy of activation of the reaction, but they themselves remain unchanged by the reaction. Thus, they are important in the regulation of biological processes, for example as activators or inhibitors. One of the most basic enzymatic reactions, first suggested by Michaelis and Menten [8], implies a substrate *S* reacting with an enzyme *E* to form a complex *SE* which in turn is converted into a product *P* and the enzyme. This is represented schematically by

(4) $$S+E\overset{k_{1}}{\underset{k_{-1}}{⇌}}\text{SE}\overset{k_{2}}{\to }P+E$$

here *k*_1_, *k*_−1_ and *k*_2_ are constant parameters associated with the rates of reaction. We denote the concentrations of the reactants by

$$C_{1}=\left[E\right],\;C_{2}=\left[S\right],\;C_{3}=\;\left[P\right],\;C_{4}=\left[\text{ES}\right].$$

Then the law of Mass Action applied to (4) leads to one equation for each reactant and hence a system of nonlinear equations. The usual approach to these equations is to assume that the initial stage of the complex *C*_4_, formation is very fast after which it is essentially at equilibrium, then we get *C*_4_ in terms of *C*_2_,

$$C_{4}=\frac{C_{1,0}C_{2}}{C_{2}+K_{M}},\;\;\;k_{M}=\frac{k_{-1}+k_{2}}{k_{1}}$$

The basic enzyme reaction model becomes then

(5) $$\left\{\begin{array}{l} \;\frac{\partial C_{1}}{\mathrm{\partial t}}-d_{1}\Delta C_{1}-m_{1}\text{div}(C_{1}\nabla \phi )=0\;\text{in}\;Q_{T}\\ \;\frac{\partial C_{2}}{\mathrm{\partial t}}-d_{2}\Delta C_{2}-m_{2}\text{div}(C_{2}\nabla \phi )=\frac{-k_{2}C_{1,0}C_{2}}{C_{2}+K_{M}}\;\text{in}\;Q_{T}\\ \;\frac{\partial C_{3}}{\mathrm{\partial t}}-d_{3}\Delta C_{3}-m_{3}\text{div}(C_{3}\nabla \phi )=\frac{k_{2}C_{1,0}C_{2}}{C_{2}+K_{M}}\;\text{in}\;Q_{T}\\ \;-\varepsilon \Delta \phi =\underset{i=1}{\overset{3}{\sum }}z_{i}C_{i}-f\;\text{in}\;Q_{T}\\ \;d_{i}\frac{\partial C_{i}}{\partial \upsilon }+m_{i}C_{i}\frac{\partial \phi }{\partial \upsilon }=0\;\text{in}\sum_{T},\text{for}i=1,2,3.\\ \;\phi (t,x)=0\;\text{in}\sum_{T}\\ \;C_{1}(0,x)=C_{1,0},\;C_{2}(0,x)=C_{2,0},\;C_{3}(0,x)=0\;\text{on}\;\Omega \\ \;\phi (0,x)=\phi_{0}(x)\;\text{on}\;\Omega \end{array}\right)$$

#### Algorithm of resolution

Before stating the resolution algorithm, we introduce the function *Z*_*h*_=(*Z*_*i*,*h*_)_1≤*i*≤3_ defined by

$$Z_{i,h}=C_{i,h}\;\text{exp}\;\left(\frac{m_{i}}{d_{i}}(\phi_{h})\right)\;\text{for}i=1,\;2,\;3$$

Moreover, we consider

$$p_{i,h}=\;\text{exp}\;\left(\frac{m_{i}}{d_{i}}\phi_{h}\right)\;\text{and}\;q_{i,h}=\frac{1}{p_{i,h}}$$

We used the following algorithm to calculate *ϕ*_*h*_ and *Z*_*i*,*h*_ then we calculate *C*_*i*,*h*_ by using the reverse relation:

$$\;C_{i,h}=\;\text{exp}\;\left(\frac{-m_{i}}{d_{i}}\phi_{h}\right)Z_{i,h}\;\;\;$$

● Initialize: for *i* = 1,2,3,

$$\begin{array}{l} Z_{i,h}^{0}=C_{i,0}(0,x)p_{i,h}(0,x),\\ q_{i,h}^{0}=\;\text{exp}\;\left(\frac{-m_{i}}{d_{i}}\phi_{h}(0,x)\right) \end{array}$$

● Loop over *n*

At step *n*: 

● Calculate $\phi_{h}^{n+1}$ solution of

$$\varepsilon \int_{\Omega }\nabla \phi_{h}^{n+1}\nabla v_{h}=\int_{\Omega }\left(z_{1}q_{1,h}^{n}Z_{1,h}^{n}+z_{2}q_{2,h}^{n}Z_{2,h}^{n}+z_{3}q_{3,h}^{n}Z_{3,h}^{n}-f\right)v_{h}$$

● Calculate $q_{1,h}^{n+1},\;q_{2,h}^{n+1},\;q_{3,h}^{n+1}$ by:

$$q_{i,h}^{n+1}=\;\text{exp}\;\left(\frac{-m_{i}}{d_{i}}\overset{n+1}{\underset{h}{\phi }}\right)$$

● Calculate $Z_{1,h}^{n+1}$ solution of

$$\int_{\Omega }\frac{q_{1,h}^{n+1}Z_{1,h}^{n+1}-q_{1,h}^{n}Z_{1,h}^{n}}{\text{dt}}w_{h}+d_{1}\int_{\Omega }q_{1,h}^{n+1}\nabla Z_{1,h}^{n+1}\nabla w_{h}=0$$

● Calculate $Z_{2,h}^{n+1}$ solution of:

Loop over *k* untill ${∥Z_{2,h}^{n+1,k+1}-Z_{2,h}^{n+1,k}∥}_{L_{2}\left(\Omega \right)}<\text{eps}$

$$\int_{\Omega }\frac{q_{2,h}^{n+1}Z_{2,h}^{n+1,k+1}-q_{2,h}^{n}Z_{2,h}^{n}}{\text{dt}}w_{h}+d_{2}\int_{\Omega }q_{2,h}^{n+1}\nabla Z_{2,h}^{n+1,k+1}\nabla w_{h}=-\int_{\Omega }\frac{k_{2}C_{1,0}q_{2,h}^{n+1}Z_{2,h}^{n+1,k+1}}{k_{M}+q_{2,h}^{n+1}Z_{2,h}^{n+1,k}}w_{h}$$

where eps is the stopping criterion. 

● Calculate $Z_{3,h}^{n+1}$solution of

$$\int_{\Omega }\frac{q_{3,h}^{n+1}Z_{3,h}^{n+1}-q_{3,h}^{n}Z_{3,h}^{n}}{\text{dt}}w_{h}+d_{3}\int_{\Omega }q_{3,h}^{n+1}\nabla Z_{3,h}^{n+1}\nabla w_{h}=\int_{\Omega }\frac{k_{2}C_{1,0}q_{2,h}^{n+1}Z_{2,h}^{n+1}}{k_{M}+q_{2,h}^{n+1}Z_{2,h}^{n+1}}w_{h}$$

#### Numerical results

Here we present changes in substrate, product and enzyme concentrations. The cell is represented by an ellipse with semi-major axis a=2 and semi-minor axis b=1. The diffusion coefficient of the ions are *d*_1_=10^−3^*m*^2^.*s*^−1^, *d*_2_=2.10^−3^*m*^2^.*s*^−1^ and *d*_3_=5.10^−3^*m*^2^.*s*^−1^. The constants of reaction are $k_{M}=9.10^{-5}\;M,k_{2}=1,4.10^{4}\;s^{-1}\text{and}k_{1}=k_{\text{cat}}=\frac{k_{2}}{k_{M}}=1,55.10^{8}\;M^{-1}.s^{-1}$. The charge number of the ions are *z*_1_=1, *z*_2_=0 and *z*_3_=1. The electric charge density is *f* = 0.1*C*. The initial conditions are *C*_1,0_=1*μM*, *C*_2,0_=800 *μM*, *C*_3,0_=0 and *ϕ*_0_=−80 *mV*; the stopping criterion is *eps* = 10^−4^. The time step of the simulation is *dt* = 10^−3^*s*.

**Figure 1:**
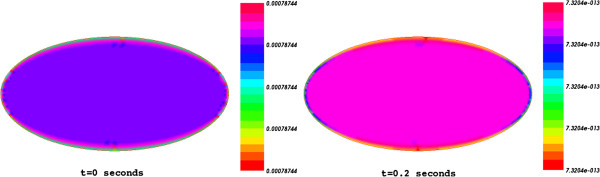
The spatial distribution of the substrate concentration through the cell at initial time t=0 and final time T=0.2 s.

Shown in Figure 1 is the spatial distribution of substrate concentration through the cell at both initial and final time (t=0 and T=200 ms).

**Figure 2:**
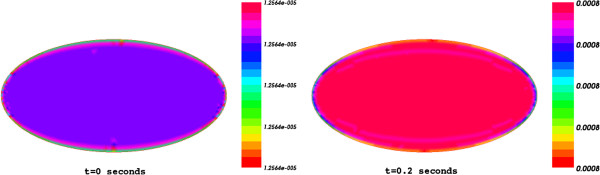
The spatial distribution of the product concentration through the cell at initial time t=0 and final time T=0.2 s.

Figure 2 presents the spatial distribution of the product concentration through the cell at both initial and final time (t=0 and T=200 ms).

**Figure 3:**
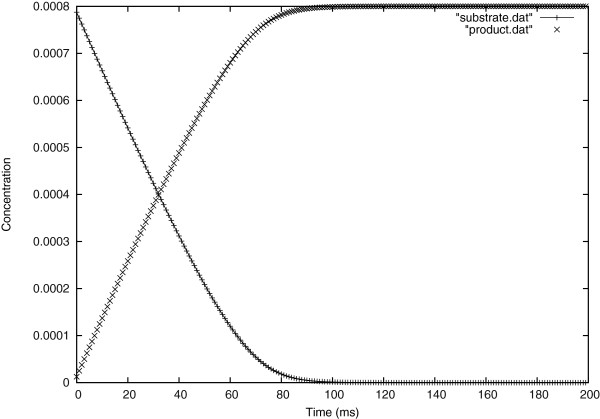
Evolution in time of substrate and product concentrations at the center of the cell.

In Figure 3, one sees the time evolution of the substrate and the product concentrations at the center of the cell. It can be seen that the substrate decrease curve is the mirror image of the product appearance curve. By observing the early times, it’s obvious that the substrate loss and product appearance change speedily with time but as time goes on these rates diminish, to reach zero when all the substrate has been converted to product by the enzyme.

**Figure 4:**
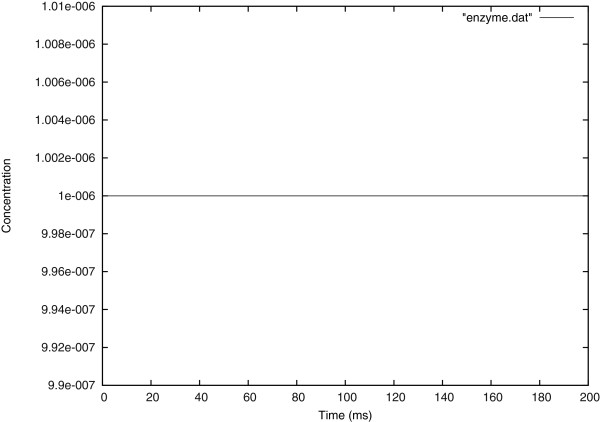
Temporal evolution of the enzyme concentration at the center of the cell.

The Figure 4 shows, as predicted, the enzyme concentration remaining constant over time.

Two simplifications of these equations have been quite popular in the literature while computing membrane reactions, firstly the Goldman hypothesis where the electrical field is supposed to be constant inside the membrane and secondly considering a system of ordinary equations depending only on time and not the space. The added value of this work is not considering all of those simplifications which leads to a more realistic model and more accurate numerical results. Moreover, the results obtained are in agreement with the experimental results found in the literature [9].

### Result 2 : Suicide substrate kinetics

An enzyme system of major experimental concern; see [10,11], is the mechanism-based inhibitor, or suicide substrate system, represented by Walsh et al. [12],

(6) $$\begin{array}{l} E+S\overset{k_{1}}{\underset{k_{-1}}{⇌}}X\overset{k_{2}}{\to }Y\overset{k_{3}}{\to }E+P\\ \;Y\overset{k_{4}}{\to }E_{i} \end{array}$$

where *E*, *S* and *P* stand for enzyme, substrate, and product, respectively; *X* and *Y*, enzyme-substrate intermediates; *E*_*i*_, inactivated enzyme; and the *k* ’s are positive rate constants.

In this system, *Y* has a choice of one of two pathways, namely, to *E*+*P* with rate *k*_3_ or to *E*_*i*_ with rate *k*_4_. The ratio of these rates, *k*_3_/*k*_4_, is called the partition ratio and is denoted by *r*. Each of these pathways are supposed to be irreversible over the timescale of the reaction see [13]. *S* is known as a suicide substrate because it binds to the active site of an enzyme—like a substrate—but the enzyme converts it into an inhibitor which irreversibly inactivates the enzyme. Thereby, the enzyme ‘commits suicide’. In this way, a suicide substrate can specifically target an enzyme for inactivation. Furthermore, suicide substrates are particularly useful in drug administration, as they are not noxious in their common form and only the designated enzyme can convert them to their inhibitor form. For example, suicide substrates have been subject of investigation for use in the treatment of depression (monoamine oxidase inhibitors, Seiler at al. [10]), epilepsy (brain GABA transaminase inhibitors, Walsh [11]), and some tumors (ornithine decarboxylase inhibitors, Seiler et al. [10]). Suicide substrate kinetics have been studied by Waley [13] and by Tatsunami et al. [14], who had interest in the factor which determined whether the substrate was exhausted before all the enzyme was inactivated. Waley proposed it was *rμ*, where *μ* is the ratio of the initial concentration of enzyme to that of substrate, namely, *e*_0_/*s*_0_. Tatsunami et al., on the other hand, found the determining factor to be (1+*r*)*μ*. When (1+*r*)*μ*>1 the substrate is exhausted, while for (1+*r*)*μ*<1, all the enzyme is inactivated. When (1+*r*)*μ* = 1, both occur. The interest is when *e*_0_/*s*_0_ is not small, which was in effect assumed since both Waley [13] and Tatsunami et al. [14] used a quasi-steady state approximation. The validity decreases for increasing values of *e*_0_/*s*_0_. We denote the concentrations of the reactants by

$$C_{1}=\left[E\right],C_{2}=\left[S\right],C_{3}=\left[X\right],C_{4}=\left[Y\right],\;C_{5}=[E_{i}],\;C_{6}=[P]$$

The law of mass action applied to (6) leads to one equation for each reactant and hence a system of nonlinear equations. We obtain the following system

(7) $$\;\;\;\left\{\begin{array}{ll} \frac{\partial C_{1}}{\mathrm{\partial t}}-d_{1}\Delta C_{1}-m_{1}\text{div}(C_{1}\nabla \phi )=-k_{1}C_{1}C_{2}+k_{-1}C_{3}+k_{3}C_{4} & \text{in}\;Q_{T}\\ \frac{\partial C_{2}}{\mathrm{\partial t}}-d_{2}\Delta C_{2}-m_{2}\text{div}(C_{2}\nabla \phi )=-k_{1}C_{1}C_{2}+k_{-1}C_{3} & \text{in}\;Q_{T}\\ \frac{\partial C_{3}}{\mathrm{\partial t}}-d_{3}\Delta C_{3}-m_{3}\text{div}(C_{3}\nabla \phi )=k_{1}C_{1}C_{2}-\left(k_{-1}+k_{2}\right)C_{3} & \text{in}\;Q_{T}\\ \frac{\partial C_{4}}{\mathrm{\partial t}}-d_{4}\Delta C_{4}-m_{4}\text{div}(C_{4}\nabla \phi )=k_{2}C_{3}-\left(k_{3}+k_{4}\right)C_{4} & \text{in}\;Q_{T}\\ \frac{\partial C_{5}}{\mathrm{\partial t}}-d_{5}\Delta C_{5}-m_{5}\text{div}(C_{5}\nabla \phi )=k_{4}C_{4} & \text{in}\;Q_{T}\\ \frac{\partial C_{6}}{\mathrm{\partial t}}-d_{6}\Delta C_{6}-m_{6}\text{div}(C_{6}\nabla \phi )=k_{3}C_{4} & \text{in}\;Q_{T}\\ -\varepsilon \Delta \phi =\overset{6}{\underset{i=1}{\sum }}z_{i}C_{i}-f & \text{in}\;Q_{T}\\ d_{i}\frac{\partial C_{i}}{\partial \upsilon }+m_{i}C_{i}\frac{\partial \phi }{\partial \upsilon }=0\;\text{in}\;\sum_{T},\text{for}i=1,2,3,\ldots ,6.\\ \phi (t,x)=0 & \text{in}\;\sum_{T}\\ \;C_{1}(0,x)=e_{0},\;C_{2}(0,x)=s_{0},\;C_{i}(0,x)=0\;\text{on}\;\Omega ,\text{for}i=3,\ldots ,6.\\ \;\phi (0,x)=\phi_{0}(x) & \text{on}\;\Omega \end{array}\right)$$

#### Algorithm of resolution

Before stating the resolution algorithm, we introduce the function *Z*_*h*_=(*Z*_*i*,*h*_)_1≤*i*≤6_ defined by

$$Z_{i,h}=C_{i,h}\;\text{exp}\;(\frac{m_{i}}{d_{i}}(\phi_{h}))\;\text{for}i=1,\;\ldots ,\;6$$

Moreover, we consider

$$p_{i,h}=\;\text{exp}\;(\frac{m_{i}}{d_{i}}\phi_{h})\;\text{and}\;q_{i,h}=\frac{1}{p_{i,h}}$$

We used the following algorithm to calculate *ϕ*_*h*_ and *Z*_*i*,*h*_ then we calculate *C*_*i*,*h*_ by using the reverse relation:

$$\;C_{i,h}=\;\text{exp}\;(\frac{-m_{i}}{d_{i}}\phi_{h})Z_{i,h}\;\;\;$$

● Initialize for *i* = 1,…,6

$$\begin{array}{ll} Z_{i,h}^{0} & =C_{i,0}(0,x)p_{i,h}(0,x),\\ \;q_{i,h}^{0} & =\;\text{exp}\;\left(\frac{-m_{i}}{d_{i}}\phi_{h}(0,x)\right)\;\;\; \end{array}$$

● Loop over *n*

At step *n*: 

● Calculate $\phi_{h}^{n+1}$ solution of

$$\begin{array}{ll} \varepsilon \int_{\Omega }\nabla \phi_{h}^{n+1}\nabla v_{h} & =\int_{\Omega }\left(\overset{6}{\underset{i=1}{\sum }}z_{i}q_{i,h}^{n}Z_{i,h}^{n}-f\right)v_{h} \end{array}$$

● Calculate $q_{1,h}^{n+1},\;q_{2,h}^{n+1},\;q_{3,h}^{n+1},\;q_{4,h}^{n+1},\;q_{5,h}^{n+1},\;q_{6,h}^{n+1}$ by:

$$q_{i,h}^{n+1}=\;\text{exp}\;\left(\frac{-m_{i}}{d_{i}}\overset{n+1}{\underset{h}{\phi }}\right)\;$$

● Calculate $Z_{1,h}^{n+1},Z_{2,h}^{n+1},Z_{3,h}^{n+1},Z_{4,h}^{n+1},Z_{5,h}^{n+1},Z_{6,h}^{n+1}$ solutions of: 

initialize $Z_{i,h}^{n+1,0}=Z_{i,h}^{n}$ for *i* = 1,…,6,

Loop over *k* untill

$$\overset{6}{\underset{i=1}{\sum }}{∥Z_{i,h}^{n+1,k+1}-Z_{i,h}^{n+1,k}∥}_{L_{2}\left(\Omega \right)}<\text{eps}$$

$$\begin{array}{lll} & \;\;\;\int_{\Omega }\frac{q_{1,h}^{n+1}Z_{1,h}^{n+1,k+1}-q_{1,h}^{n}Z_{1,h}^{n}}{\text{dt}}w_{h}+d_{1}\int_{\Omega }q_{1,h}^{n+1}\nabla Z_{1,h}^{n+1,k+1}\nabla w_{h}\; & \;\\ & =\int_{\Omega }\left(-k_{1}q_{2,h}^{n+1}Z_{2,h}^{n+1,k}q_{1,h}^{n+1}Z_{1,h}^{n+1,k+1}+k_{-1}q_{3,h}^{n+1}Z_{3,h}^{n+1,k}+k_{3}q_{4,h}^{n+1}Z_{4,h}^{n+1,k}\right)w_{h}\; & \;\\ & \;\;\;\int_{\Omega }\frac{q_{2,h}^{n+1}Z_{2,h}^{n+1,k+1}-q_{2,h}^{n}Z_{2,h}^{n}}{\text{dt}}w_{h}+d_{2}\int_{\Omega }q_{2,h}^{n+1}\nabla Z_{2,h}^{n+1,k+1}\nabla w_{h}\; & \;\\ & =\int_{\Omega }\left(-k_{1}q_{2,h}^{n+1}Z_{2,h}^{n+1,k+1}q_{1,h}^{n+1}Z_{1,h}^{n+1,k+1}+k_{-1}q_{3,h}^{n+1}Z_{3,h}^{n+1,k}\right)w_{h}\; & \; \end{array}$$

$$\begin{array}{lll} & \;\;\;\int_{\Omega }\frac{q_{3,h}^{n+1}Z_{3,h}^{n+1,k+1}-q_{3,h}^{n}Z_{3,h}^{n}}{\text{dt}}w_{h}+d_{3}\int_{\Omega }q_{3,h}^{n+1}\nabla Z_{3,h}^{n+1,k+1}\nabla w_{h}\; & \;\\ & =\int_{\Omega }\left(k_{1}q_{2,h}^{n+1}Z_{2,h}^{n+1,k+1}q_{1,h}^{n+1}Z_{1,h}^{n+1,k+1}-\left(k_{-1}+k_{2}\right)q_{3,h}^{n+1}Z_{3,h}^{n+1,k+1}\right)w_{h}\;\; & \; \end{array}$$

$$\begin{array}{l} \;\;\;\int_{\Omega }\frac{q_{4,h}^{n+1}Z_{4,h}^{n+1,k+1}-q_{4,h}^{n}Z_{4,h}^{n}}{\text{dt}}w_{h}+d_{4}\int_{\Omega }q_{4,h}^{n+1}\nabla Z_{4,h}^{n+1,k+1}\nabla w_{h}\\ =\int_{\Omega }\left(k_{2}q_{3,h}^{n+1}Z_{3,h}^{n+1,k+1}-\left(k_{3}+k_{4}\right)q_{4,h}^{n+1}Z_{4,h}^{n+1,k+1}\;\right)w_{h}\; \end{array}$$

$$\int_{\Omega }\frac{q_{5,h}^{n+1}Z_{5,h}^{n+1,k+1}-q_{5,h}^{n}Z_{5,h}^{n}}{\text{dt}}w_{h}+d_{5}\int_{\Omega }q_{5,h}^{n+1}\nabla Z_{5,h}^{n+1,k+1}\nabla w_{h}=\int_{\Omega }k_{4}q_{4,h}^{n+1}Z_{4,h}^{n+1,k+1}w_{h}\;$$

$$\int_{\Omega }\frac{q_{6,h}^{n+1}Z_{6,h}^{n+1,k+1}-q_{6,h}^{n}Z_{6,h}^{n}}{\text{dt}}w_{h}+d_{6}\int_{\Omega }q_{6,h}^{n+1}\nabla Z_{6,h}^{n+1,k+1}\nabla w_{h}=\int_{\Omega }k_{3}q_{4,h}^{n+1}Z_{4,h}^{n+1,k+1}w_{h}$$

#### Numerical results

Here we present changes in substrate, product, enzyme, inactivated enzyme and the intermediate concentrations (*X* and *Y*). The cell is represented by an ellipse with semi-major axis a=2 and semi-minor axis b=1. The diffusion coefficients of the ions are *d*_1_=10^−3^*m*^2^.*s*^−1^, *d*_2_=2.10^−3^*m*^2^.*s*^−1^, *d*_3_=5.10^−3^*m*^2^.*s*^−1^, *d*_4_=10^−3^*m*^2^. *s*^−1^, *d*_5_=2.10^−3^*m*^2^.*s*^−1^, *d*_6_=4.10^−6^*m*^2^.*s*^−1^, the reaction parameters are *k*_1_=2 *s*^−1^, *k*_−1_=4 *s*^−1^, *k*_2_=12 *s*^−1^, *k*_3_=10 *s*^−1^ and *k*_4_=2 *s*^−1^. The charge number of the ions are *z*_1_=1, *z*_2_=0, *z*_3_=1, *z*_4_=1, *z*_5_=1 and *z*_6_=0. The electric charge density is *f* = 0.1*C*. The initial concentrations are *e*_0_=0.5*μM* and *s*_0_=0.5*μM*;and *ϕ*_0_=−80 *mV*. The time step of the simulation is *dt* = 10^−2^*s*. The data employed for the reaction parameters and initial concentrations were taken from Burke et al. [15].

**Figure 5:**
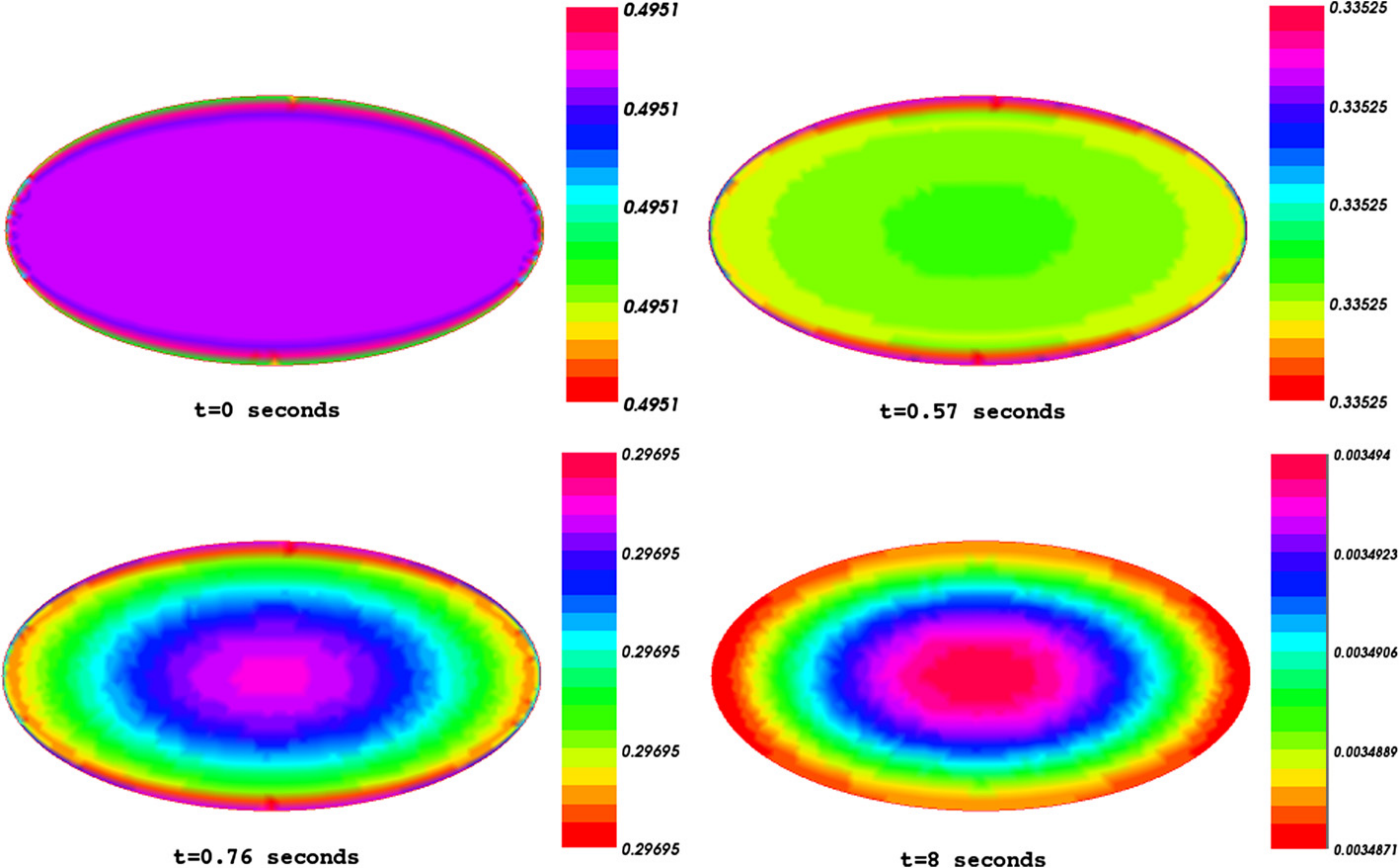
Substrate concentration from initial time to final time.

Figure 5 plots the changes in the concentration distribution of substrate from initial time to final time. One can see that in final time the substrate was totally exhausted. This complete consumption of the substrate is in agreement with the prediction of Tatsunami et al. [14] as (1+*r*)*μ* = 6>1.

**Figure 6:**
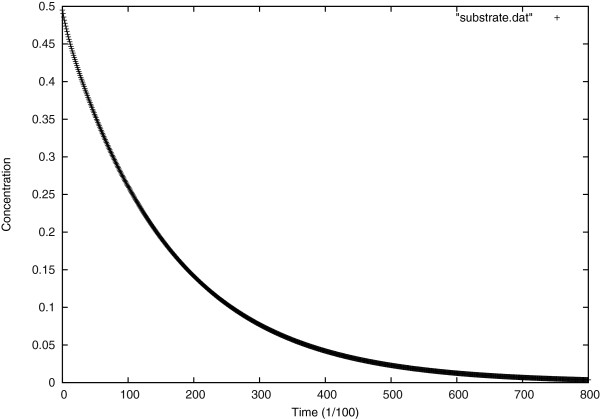
Evolution of substrate concentration over time at the center of the cell.

In Figure 6, one sees the evolution of substrate concentration over time at the center of the specimen.

**Figure 7:**
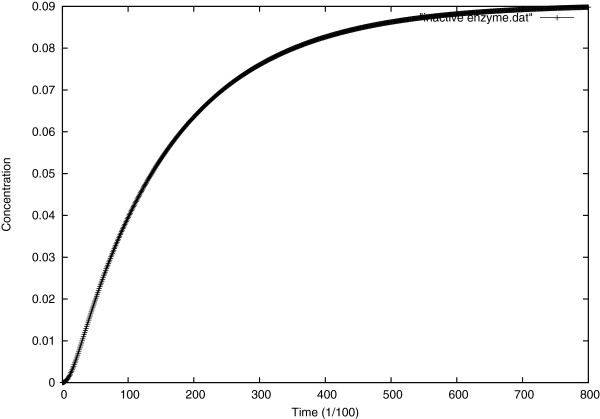
Evolution of inactivated enzyme concentration over time at the center of the cell.

Figure 7 shows the evolution of inactivated enzyme concentration over time at the center of the specimen.

**Figure 8:**
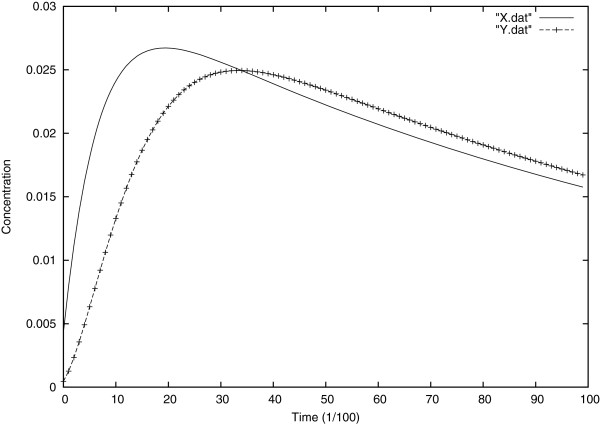
**Evolution of the intermediate concentrations *X* and *Y *over time at the center of the cell.**

Figure 8 shows the numerical solutions for intermediate concentrations of *X* and *Y*.

**Figure 9:**
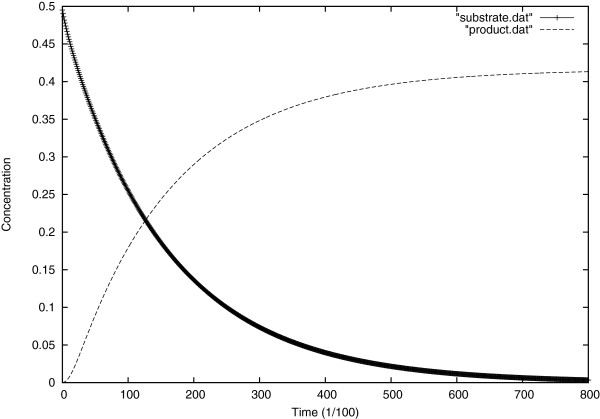
Evolution of substrate and product concentrations over time.

In Figure 9, is represented the graphic of the evolution of substrate and product at the center of the cell, comparing that result with Figure 3 (Michaelis and Menten model), here the two plots are asymmetric which is logical as we know that an amount of the substrate instead of being converted to product, is forming the inactivated enzyme (inactivating the enzyme).

**Figure 10:**
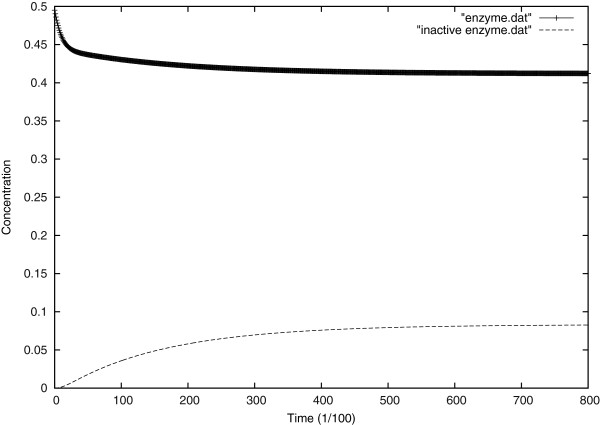
Evolution of enzyme and inactivated enzyme over time at the center of the cell.

To illustrate, Figure 10 shows the decrease in the enzyme concentration unlike the Michaelis Menten model (Figure 4); however, as the intermediate enzymes X and Y, vanish in few milliseconds, we see the loss in enzyme compensated by the production of the inactivated enzyme: the enzyme commits suicide.

To highlight the accuracy of these results, we compared them first with the numerical solutions and the approximate asymptotic solutions obtained both by Burke et al. [15], they considered a system of ordinary differential equations depending on time as they neglected the spatial aspect of the biochemical reaction, and they supposed that the electrical potential inside the membrane remains constant. For the numerical solutions Burke et al. [15] solved the system numerically, but for the approximate asymptotic solutions they non-dimensionalise the same system, and used asymptotic methods and a method detailed in Kevorkian and Cole [16]. Finally we compared our results with previous approximate methods of Tatsunami et al. [14] and Waley [17] which were based on a pseudo-steady state hypothesis. This comparison shows that the results described here are valid numerical solutions for the kinetics of suicide substrate system. The solution for the substrate and inactivated enzyme are more accurate than those of previous approximations [14,17], especially in small time, which is by definition ignored by any pseudo-steady-state approximate method. Furthermore, the method presented here is specially useful in estimating the intermediate (*X* and *Y*) concentrations besides incorporating the spatial and the electro-migration aspects of the phenomena.

### Result 3 : Cooperative phenomena

An enzyme reaction is said to be cooperative if a single enzyme molecule, after binding a substrate molecule at one site can then bind another substrate molecule at another site. Such phenomena are quite common in living organisms. Another interesting cooperative reaction is when an enzyme with several binding sites is such that the binding of one substrate molecule at one site can affect the activity of binding other substrate molecules at another site. This indirect interaction between distinct and specific binding sites is called allostery, and an enzyme displaying it, an allosteric enzyme. When a substrate that binds at one site increases the binding activity at another site then the substrate is called an activator, otherwise (if it decreases the activity) it’s called an inhibitor.

As an example of cooperative phenomenon we consider the case when an enzyme has two binding sites. A model for this consists of an enzyme molecule *E* which binds a substrate molecule *S* to form a single bound substrate-enzyme complex *X*. This complex *X* not only breaks down to form a product *P* and the enzyme *E* again, it can also combine with another substrate molecule to form a dual bound substrate-enzyme complex *Y*. This *Y* complex breaks down to form the product *P* and the single bound complex *X*. The reaction mechanism is represented schematically by

(8) $$\begin{array}{ll} S+E & \overset{k_{1}}{\underset{k_{-1}}{⇌}}X\overset{k_{2}}{\to }E+P\\ S+X & \overset{k_{3}}{\underset{k_{-3}}{⇌}}Y\overset{k_{4}}{\to }X+P \end{array}$$

Here *k*^′^*s* are rate constants. We denote the concentrations of the reactants by

$$C_{1}=\left[E\right],\;C_{2}=\left[S\right],C_{3}=\left[X\right],\;C_{4}=\left[Y\right],\;C_{5}=\left[P\right].$$

Then the law of Mass Action applied to (8) leads to one equation for each reactant and hence the system of nonlinear reaction. We have then

(9) $$\left\{\begin{array}{ll} \frac{\partial C_{1}}{\mathrm{\partial t}}-d_{1}\Delta C_{1}-m_{1}\text{div}(C_{1}\nabla \phi )=-k_{1}C_{2}C_{1}+\left(k_{-1}+k_{2}\right)C_{3} & \text{in}\;Q_{T}\\ \frac{\partial C_{2}}{\mathrm{\partial t}}-d_{2}\Delta C_{2}-m_{2}\text{div}(C_{2}\nabla \phi )=-k_{1}C_{2}C_{1}+\left(k_{-1}-k_{3}C_{2}\right)C_{3}+k_{-3}C_{4} & \text{in}\;Q_{T}\\ \frac{\partial C_{3}}{\mathrm{\partial t}}-d_{3}\Delta C_{3}-m_{3}\text{div}(C_{3}\nabla \phi )=k_{1}C_{2}C_{1}-\left(k_{-1}+k_{2}+k_{3}C_{2}\right)C_{3}+(k_{-3}+k_{4})C_{4} & \text{in}\;Q_{T}\\ \frac{\partial C_{4}}{\mathrm{\partial t}}-d_{4}\Delta C_{4}-m_{4}\text{div}(C_{4}\nabla \phi )=k_{3}C_{2}C_{3}-(k_{-3}+k_{4})C_{4} & \text{in}\;Q_{T}\\ \frac{\partial C_{5}}{\mathrm{\partial t}}-d_{5}\Delta C_{5}-m_{5}\text{div}(C_{5}\nabla \phi )=k_{2}C_{3}+k_{4}C_{4} & \text{in}\;Q_{T}\\ -\varepsilon \Delta \phi =\overset{5}{\underset{i=1}{\sum }}z_{i}C_{i}-f & \text{in}Q_{T}\\ d_{i}\frac{\partial C_{i}}{\partial \upsilon }+m_{i}C_{i}\frac{\partial \phi }{\partial \upsilon }=0\;\text{in}\;\sum_{T},\text{for}i=1,\ldots ,5\\ \phi (t,x)=0 & \text{in}\;\sum_{T}\\ \;C_{1}(0,x)=e_{0},\;C_{2}(0,x)=s_{0},\;C_{i}(0,x)=0\;\text{on}\;\Omega ,\;\text{for}\;i=3,4,5.\\ \phi (0,x)=\phi_{0}(x) & \text{on}\;\Omega \end{array}\right)$$

#### Algorithm of resolution

Before stating the resolution algorithm, we introduce the function *Z*_*h*_=(*Z*_*i*,*h*_)_1≤*i*≤5_ defined by

$$Z_{i,h}=C_{i,h}\;\text{exp}\;\left(\frac{m_{i}}{d_{i}}(\phi_{h})\right)\;\text{for}i=1,\;\ldots \;,\;5\;$$

Moreover, we consider

$$p_{i,h}=\;\text{exp}\;\left(\frac{m_{i}}{d_{i}}\phi_{h}\right)\;\text{and}\;q_{i,h}=\frac{1}{p_{i,h}}$$

We used the following algorithm to calculate *ϕ*_*h*_ and *Z*_*i*,*h*_ then we calculate *C*_*i*,*h*_ by using the reverse relation:

$$\;C_{i,h}=\;\exp\;\left(\frac{-m_{i}}{d_{i}}\phi_{h}\right)Z_{i,h}\;\;\;$$

● Initialize for *i* = 1,…,5

● 

$$\begin{array}{ll} Z_{i,h}^{0} & =C_{i,0}(0,x)p_{i,h}(0,x),\\ q_{i,h}^{0} & =\exp\left(\frac{-m_{i}}{d_{i}}\phi_{h}(0,x)\right)\;\;\; \end{array}$$

● Loop over *n*

● At step *n*:

● Calculate $\phi_{h}^{n+1}$ solution of

$$\begin{array}{ll} \varepsilon \int_{\Omega }\nabla \phi_{h}^{n+1}\nabla v_{h} & =\int_{\Omega }\left(\sum_{i=1}^{5}z_{i}q_{i,h}^{n}Z_{i,h}^{n}-f\right)v_{h} \end{array}$$

● Calculate $q_{1,h}^{n+1},\;q_{2,h}^{n+1},\;q_{3,h}^{n+1},\;q_{4,h}^{n+1},\;q_{5,h}^{n+1}$ by:

$$q_{i,h}^{n+1}=\exp\left(\frac{-m_{i}}{d_{i}}\overset{n+1}{\underset{h}{\phi }}\right)\;$$

● Calculate $Z_{1,h}^{n+1},Z_{2,h}^{n+1},Z_{3,h}^{n+1},Z_{4,h}^{n+1},Z_{5,h}^{n+1}$ solutions of: 

initialize $Z_{i,h}^{n+1,0}=Z_{i,h}^{n}$ for *i* = 1,…,5,

Loop over *k* untill

$$\overset{5}{\underset{i=1}{\sum }}{∥Z_{i,h}^{n+1,k+1}-Z_{i,h}^{n+1,k}∥}_{L_{2}\left(\Omega \right)}<\text{eps}$$

$$\begin{array}{lll} \; & \;\;\;\int_{\Omega }\frac{q_{1,h}^{n+1}Z_{1,h}^{n+1,k+1}-q_{1,h}^{n}Z_{1,h}^{n}}{\text{dt}}w_{h}+d_{1}\int_{\Omega }q_{1,h}^{n+1}\nabla Z_{1,h}^{n+1,k+1}\nabla w_{h}\; & \;\\ \; & =\int_{\Omega }\left(-k_{1}q_{2,h}^{n+1}Z_{2,h}^{n+1,k}q_{1,h}^{n+1}Z_{1,h}^{n+1,k+1}+(k_{-1}+k_{2})q_{3,h}^{n+1}Z_{3,h}^{n+1,k}\right)w_{h}\; & \; \end{array}$$

$$\begin{array}{lll} \; & \;\;\;\int_{\Omega }\frac{q_{2,h}^{n+1}Z_{2,h}^{n+1,k+1}-q_{2,h}^{n}Z_{2,h}^{n}}{\text{dt}}w_{h}+d_{2}\int_{\Omega }q_{2,h}^{n+1}\nabla Z_{2,h}^{n+1,k+1}\nabla w_{h}\; & \;\\ \; & =\int_{\Omega }\left(-k_{1}q_{2,h}^{n+1}Z_{2,h}^{n+1,k+1}q_{1,h}^{n+1}Z_{1,h}^{n+1,k+1}+(k_{-1}-k_{3}q_{2,h}^{n+1}Z_{2,h}^{n+1,k+1})q_{3,h}^{n+1}Z_{3,h}^{n+1,k}\right)w_{h}\; & \;\\ \; & \;+\int_{\Omega }k_{-3}q_{4,h}^{n+1}Z_{4,h}^{n+1,k}w_{h}\; & \; \end{array}$$

$$\begin{array}{lll} \; & \;\;\;\int_{\Omega }\frac{q_{3,h}^{n+1}Z_{3,h}^{n+1,k+1}-q_{3,h}^{n}Z_{3,h}^{n}}{\text{dt}}w_{h}+d_{3}\int_{\Omega }q_{3,h}^{n+1}\nabla Z_{3,h}^{n+1,k+1}\nabla w_{h}\; & \;\\ \; & =\int_{\Omega }\left(k_{1}q_{2,h}^{n+1}Z_{2,h}^{n+1,k+1}q_{1,h}^{n+1}Z_{1,h}^{n+1,k+1}-(k_{-1}+k_{2}+k_{3}q_{2,h}^{n+1}Z_{2,h}^{n+1,k+1})q_{3,h}^{n+1}Z_{3,h}^{n+1,k+1}\right)w_{h}\; & \;\\ \; & \;+\int_{\Omega }\left((k_{-3}+k_{4})q_{4,h}^{n+1}Z_{4,h}^{n+1,k}\right)w_{h}\; & \; \end{array}$$

$$\begin{array}{lll} \; & \;\;\;\int_{\Omega }\frac{q_{4,h}^{n+1}Z_{4,h}^{n+1,k+1}-q_{4,h}^{n}Z_{4,h}^{n}}{\text{dt}}w_{h}+d_{4}\int_{\Omega }q_{4,h}^{n+1}\nabla Z_{4,h}^{n+1,k+1}\nabla w_{h}\; & \;\\ \; & =\int_{\Omega }\left(k_{3}q_{2,h}^{n+1}Z_{2,h}^{n+1,k+1}q_{3,h}^{n+1}Z_{3,h}^{n+1,k+1}-(k_{-3}+k_{4})q_{4,h}^{n+1}Z_{4,h}^{n+1,k+1}\right)\;w_{h}\; & \; \end{array}$$

$$\begin{array}{lll} \; & \;\;\;\int_{\Omega }\frac{q_{5,h}^{n+1}Z_{5,h}^{n+1,k+1}-q_{5,h}^{n}Z_{5,h}^{n}}{\text{dt}}w_{h}+d_{5}\int_{\Omega }q_{5,h}^{n+1}\nabla Z_{5,h}^{n+1,k+1}\nabla w_{h}\; & \;\\ \; & =\int_{\Omega }\left(k_{2}q_{3,h}^{n+1}Z_{3,h}^{n+1,k+1}+k_{4}q_{4,h}^{n+1}Z_{4,h}^{n+1,k+1}\right)w_{h}\; & \; \end{array}$$

#### Numerical results

Here, we present the evolution of product and enzyme concentrations over time in both positive and negative cooperativity. We used the same constant parameters as the previous example for the diffusion coefficients of the species and the same initial conditions. The cell is represented by an ellipse with semi-major axis a=2 and semi-minor axis b=1. To ensure cooperativity, the positive rate constants are chosen by the following reasoning: Suppose that the binding of the first substrate molecule is slow, but that with one site bound, binding of the second is fast (this is large cooperativity). This can be modeled by letting *k*_3_→*∞* and *k*_1_→0 while keeping *k*_1_*k*_3_ constant, in which case *K*_2_→0 and *K*_1_→*∞* while *K*_2_*K*_1_ is constant (*K*_1_ and *K*_2_ were introduced as they appear in the expression of velocity reaction, this is discussed in details in the book by Keener and Sneyd [18]), where

$$\begin{array}{ll} K_{1} & =\frac{k_{-1}+k_{2}}{k_{1}}\\ K_{2} & =\frac{k_{4}+k_{-3}}{k_{3}}. \end{array}$$

An enzyme can also exhibit negative cooperativity, in which the binding of the first substrate molecule decreases the rate of subsequent binding. This can be modeled by decreasing *k*_3_. We used for positive cooperativity *K*_1_=1000, *K*_2_=0.001 and *K*_1_=0.5, *K*_2_=100 for negative cooperativity (this values were taken from [18]).

**Figure 11:**
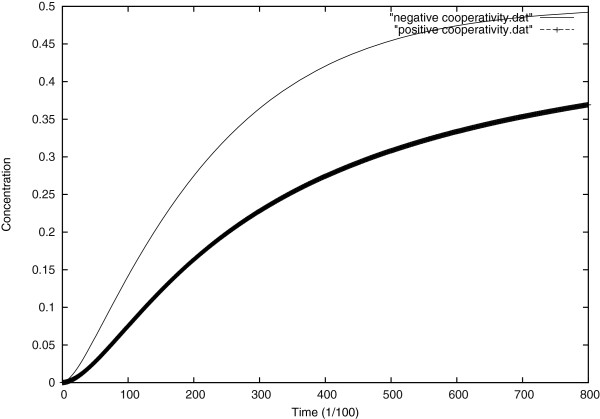
Evolution of the product concentration in both positive and negative cooperativity.

In Figure 11, one sees that in positive cooperative reaction, the product concentration is characterized by an “S-shaped” sigmoidal curve, which is different from other enzyme reaction that exhibits a curve that tends to be hyperbolic. This results from cooperative effects; in which the enzyme can bind more than one substrate molecule, but the binding of one substrate molecule affects the binding of subsequent one.

**Figure 12:**
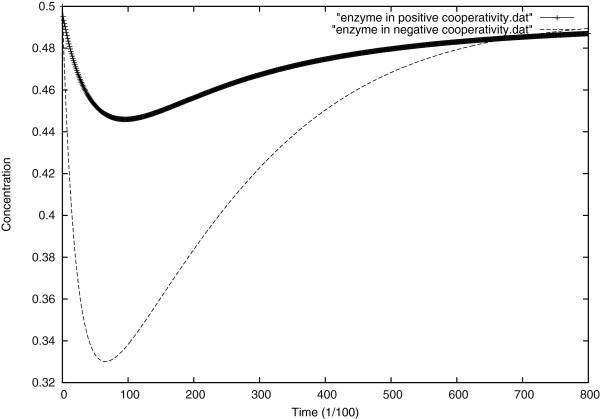
Evolution of the enzyme concentration in both positive and negative cooperativity.

In Figure 12, we plot the evolution of the enzyme concentration for both extreme positive cooperativity and negative cooperativity.

## The convergence test

The rate of convergence of the scheme is difficult to prove analytically. However, numerical experimentation suggests that the scheme is second-order accurate in space. A quantitative estimate of the convergence error was obtained by performing a number of simulations for the same initial condition on a set of increasingly finer space meshes and time steps. The initial conditions are constants. Let *T*_*h*_ the mesh generation of *Ω*, and *h*(*T*_*h*_)=*max*{*diam*(*e*_*k*_)|*e*_*k*_∈*T*_*h*_}, we take *h* = 0.3,*h* = 0.1 and *h* = 0.05. For each mesh we integrate to time *T* with $\text{dt}=\frac{\text{hT}}{16}$. Note that as we refine the space step we also refine the time step. The error of the numerical solution was defined as

$$E\left(h\right)=\text{dt}\times \overset{N_{s}}{\underset{i=1}{\sum }}\overset{N_{t}}{\underset{n=0}{\sum }}\underset{k}{\max}{∥Z_{i,h}^{n,k+1}-Z_{i,h}^{n,k}∥}_{L^{2}\left(\Omega \right)}$$

### The convergence of the basic enzyme reaction

In Table 1 is presented the error of convergence for different mesh sizes in the case of basic enzyme reaction.

**Table 1:** Convergence results for the basic enzyme reaction

**Mesh size**	***h***_**1**_** = 0.3**	***h***_**2**_** = 0.1**	***h***_**3**_** = 0.05**
Error	268.10^−7^	72.10^−7^	45.10^−7^

### The convergence of the suicide substrate reaction

In Table 2 is presented the error of convergence for different mesh sizes in the case of suicide substrate reaction.

**Table 2:** Convergence results for the suicide substrate reaction

**Mesh size**	***h***_**1**_** = 0.3**	***h***_**2**_** = 0.1**	***h***_**3**_** = 0.05**
Error	93.10^−3^	22.10^−3^	56.10^−5^

### The convergence of the cooperative reaction

In Table 3 is presented the error of convergence for different mesh sizes in the case of cooperative reaction.

**Table 3:** Convergence results for the cooperative reaction

**Mesh size**	***h***_**1**_** = 0.3**	***h***_**2**_** = 0.1**	***h***_**3**_**= 0.05**
Error	49.10^−4^	28.10^−4^	93.10^−5^

## Stability and accuracy tests

Now, let us give some information about the numerical stability of our algorithms. We perform a numerical experiment with different time step *dt*, $\frac{\text{dt}}{2}$ and $\frac{\text{dt}}{4}$. These results suggest that the scheme is indeed unconditionally stable as the solutions are quasi the same for different time steps. To illustrate, we chose to represent the product concentration.

### Stability of the basic enzyme reaction

**Figure 13:**
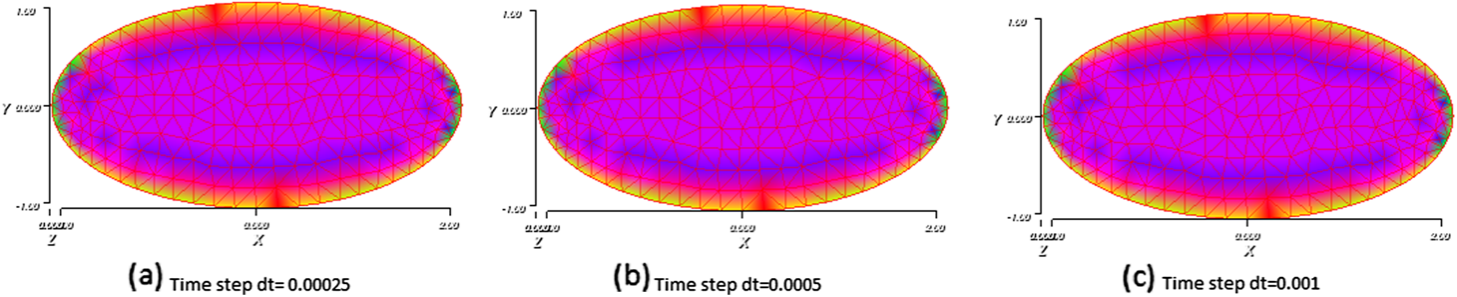
**Snapshots of the product concentration in the basic reaction at T=0.5 with three different time steps, shown in subfigures (a), (b) and (c).** The time steps are given below each subfigure.

In Figure 13, We display snapshots of the product concentration at time T=0.5 with three different time steps dt=0.00025, dt=0.0005 and dt=0.001. We can see that the results are quasi the same at the final time T.

### Stability of the suicide substrate reaction

**Figure 14:**
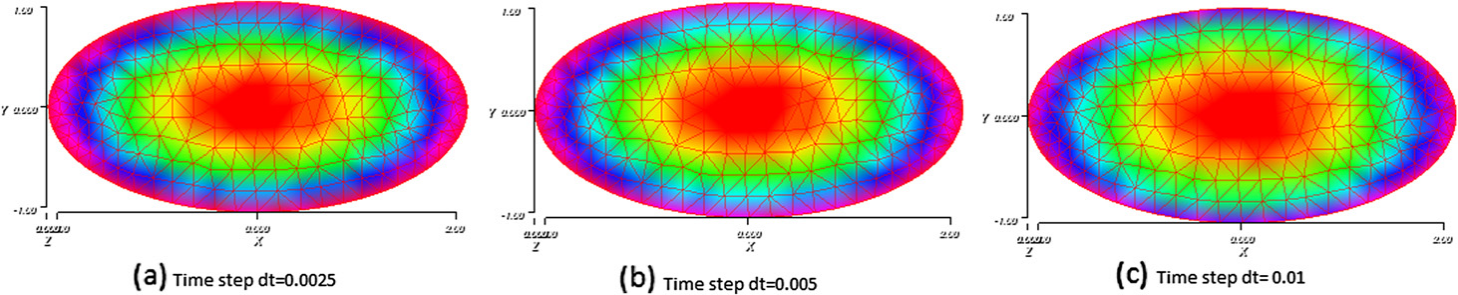
**Snapshots of the product concentration in the suicide substrate reaction at T=4 with three different time steps, shown in subfigures (a), (b) and (c).** The time steps are given below each subfigure.

In Figure 14, we display snapshots of the product concentration at time T=4 with three different time steps dt=0.0025, dt=0.005 and dt=0.01. We can see that the results are quasi the same at the final time T.

### Stability of the cooperative reaction

**Figure 15:**
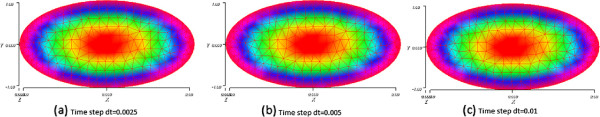
**Snapshots of the product concentration in the cooperative reaction at T=4 with three different time steps, shown in subfigures (a), (b) and (c).** The time steps are given below each subfigure.

Figure 15 shows snapshots of the product concentration at time T=4 with three different time steps dt=0.0025, dt=0.005 and dt=0.01. We can see that the results are quasi the same at the final time T.

## Conclusion

In this paper, a new model simulating ions electro-migration through biological membranes is proposed by using a more general mathematical model and a numerical technique based on the finite element method. The results presented here demonstrate that the model’s behavior agrees with the behavior of biochemical reactions as it’s consistent with the physical interpretation of the phenomena. Moreover, after comparison we can observe a complete consistency with literature findings [9,13-15,18]. A variety of numerical experiments were presented to confirm the accuracy, efficiency, and stability of the proposed method. In particular, the scheme was shown to be unconditionally stable and second-order accurate in space.

## Competing interests

The authors declare they have no competing interests.

## Authors’ contributions

Both authors contributed to writing and improving the paper and approved the final manuscript.
